# Mycophenolate mofetil prevents high-fat diet–induced hypertension and renal glomerular injury in Dahl SS rats

**DOI:** 10.1002/phy2.137

**Published:** 2013-11-05

**Authors:** Frank T Spradley, Carmen De Miguel, Janet Hobbs, David M Pollock, Jennifer S Pollock

**Affiliations:** Section of Experimental Medicine, Department of Medicine, Medical College of Georgia, Georgia Regents UniversityAugusta, 30912, Georgia

**Keywords:** Dahl salt-sensitive rat, glomerulus, high-fat diet, hypertension, SS-13^BN^ rat, T lymphocytes

## Abstract

We designed experiments to test the hypothesis that Dahl salt-sensitive (SS) rats are sensitive to high-fat diet (HFD)–induced hypertension and renal injury via an inflammatory mechanism. Twelve-week-old Dahl SS rats were maintained on a normal diet (ND; 14% fat), HFD (59% fat), or HFD supplemented with the lymphocyte immunosuppressive agent, mycophenolate mofetil (HFD + MMF; 30 mg/kg/day orally in diet), for a period of 4 weeks. Mean arterial pressure (MAP), metabolic parameters, T lymphocyte (CD3^+^) localization, and renal structural damage were assessed during the studies. Four weeks of HFD significantly elevated MAP and visceral adiposity without changing circulating levels of lipids or adipokines. Immunohistochemical analysis demonstrated that SS rats on HFD had significantly greater numbers of CD3^+^ cells in renal glomerular and medullary areas compared to ND SS rats. Additionally, HFD led to increased glomerular injury, but did not alter renal medullary injury. Chronic MMF treatment in HFD-fed Dahl SS rats reduced MAP, visceral adiposity, infiltration of CD3^+^ cells in the glomerulus, as well as glomerular injury. However, MMF treatment did not alter HFD-induced infiltration of CD3^+^ cells in the renal medulla. In conclusion, Dahl SS rats are sensitized to HFD-induced hypertension and renal glomerular injury via infiltration of T lymphocytes.

## Introduction

The two major diet-related lifestyle modifications recommended by the American Heart Association to lower blood pressure are reduced salt intake and weight loss (with a desirable body mass index <25 kg/m^2^) (Appel et al. 2011). Human studies have suggested a genetic component in the blood pressure responsiveness to increased dietary salt and weight gain (Grim et al. 1980; Egan et al. 1989; Weinberger 1996; Agapitov et al. 2008). Previous reports suggest that patients with salt-sensitive (SS) hypertension also have a greater blood pressure response to weight gain (Okosun et al. 2006; Kotchen et al. 2008). The Centers for Disease Control and Prevention reported that hypertensive renal injury is not declining and is the ninth cause of death in the United States (Molony and Craig 2009).

Studies in experimental animal models have yielded insight into mechanisms of diet-induced renal injury. Classical studies using the Dahl SS rat have demonstrated that this rat strain mimics the genetic predisposition to SS hypertension seen in some human populations, especially African Americans. It is well defined in SS rats that high-salt diet induces dramatic hypertensive renal injury associated with glomerulosclerosis, deposition of proteinaceous casts in the renal tubules, and increased albuminuria (Johnson et al. 2000; Siegel et al. 2004; Dahly-Vernon et al. 2005; Mori et al. 2008), as well as increased renal infiltration of T lymphocytes (Mattson et al. 2006; De Miguel et al. 2011a). Immunosuppressive therapy with mycophenolate mofetil (MMF) reduces renal lymphocytes, hypertension, and renal pathologies in SS rats on a high-salt diet independent of any direct hemodynamic effect of MMF (Mattson et al. 2005; Kotchen et al. 2008).

More recent reports using the SS rat have highlighted a genetic component of blood pressure and renal injury responsiveness to elevated dietary fat as well. Indeed, reports show that SS rats are hypertensive after only 4 weeks on a high-fat diet (HFD) at normal salt dietary levels (Mattson et al. 2005; Nagae et al. 2009). This is in contrast to the several months of HFD feeding needed to increase blood pressure and to induce renal injury and dysfunction in other rodent strains (Hall et al. 1993; Dobrian et al. 2000; do Carmo et al. 2009; Ma et al. 2011). Indeed, it has been shown that 5 months of HFD intake in SS rats elicits hypertension and renal injury with striking albuminuria compared to SS rats maintained on a normal diet (ND) and their genetic control strain, the SS-13^BN^ rat, which does not present with hypertension or renal injury in response to HFD (Beyer et al. 2012). These studies did not elucidate mechanisms by which HFD may lead to hypertension and renal injury in SS rats. We hypothesized that HFD-induced hypertension and renal damage are dependent on lymphocyte inflammatory mechanisms.

## Methods

### Animals

Dahl SS rats were bred in-house at Georgia Regents University from purchased SS breeders (Charles River Laboratories, Wilmington, MA). SS-13^BN^ genetic control rats were purchased from Charles River Laboratories and arrived at our institution at 10–11 weeks old. All animal experiments were in accordance with the National Institutes of Health *Guide for the Care and Use of Laboratory Animals* with all animal use protocols approved by the Georgia Regents University Institutional Animal Care and Use Committee. All rats were maintained on Harlan Teklad 8604 ND (Teklad, Madison, WI) and water was provided ad libitum. The Teklad diet or ND consisted of 3.93 kcal/g of gross energy with calories from: 33% protein, 53% carbohydrates, and 14% fat; this diet contains 0.4% NaCl. At 12 weeks old, SS and SS-13^BN^ rats were randomly placed on a HFD (F2685; BioServ, Frenchtown, NJ) or remained on ND for 4 weeks. The HFD consisted of 5.45 kcal/g of gross energy with calories from: 15% protein, 27% carbohydrates, and 59% fat. This diet contains 0.8% NaCl. A subset of HFD rats were treated with the immunosuppressant MMF for the same period of time they were on HFD (HFD+MMF, 30 mg/kg/day, orally in diet). At the end of the study at 16 weeks old, body weights were measured and rats were placed in metabolic cages to collect daily food intake and 24 h urine volumes. Rats were euthanized under pentobarbital sodium (Nembutal; 0.5 mg/kg; Abbott Laboratories, Abbott Park, IL) anesthesia. Epididymal adiposity was assessed and kidneys were isolated and prepared for histological studies as previously described (Schneider et al. 2010). Blood was collected in ethylenediaminetetraacetic acid (Sigma, St. Loius, MO)-primed syringes, spun at 3000 × g for 10 min, and snap frozen in liquid N_2_.

### Biotelemetry

A subset of rats was implanted with telemetry transmitters (Data Sciences International; St. Paul, MN) at 11 weeks old as described previously (Elmarakby et al. 2005). Rats recovered from surgery ∼1 week while having free access to tap water and ND. From 12–16 weeks old, rats were fed ND, HFD, or HFD + MMF ad libitum, while mean arterial blood pressure measurements were collected every tenth minute. Blood pressure is reported as a 24 h average. Heart rate and activity are reported as a 24 h average at 16 weeks old.

### Plasma and urine assays

Plasma (1 mL) was injected onto a Superose 6 10/300 gel filtration column (GE Healthcare Bio-Sciences AB, Uppsala, Sweden) for separation of low density lipoprotein, very low density lipoprotein, and high density lipoprotein via fast protein liquid chromatography (ÄKTA; GE) as described by Jiao et al. (1990). Fractions (0.6 mL) were collected and lipoproteins determined by colorimetric assay (TCho E; Wako Diagnostics, Osaka, Japan). Total cholesterol was defined as the sum of all lipoprotein fractions. Plasma triglycerides (Cayman Chemicals, Ann Arbor, MI), free fatty acids (Zen-Bio, Research Triangle Park, NC), and leptin (Cayman Chemicals) were assessed by enzyme-linked immunoabsorbent assay (ELISA) according to manufacturer's specifications. Plasma renin activity (PRA) was determined via radioimmunoassay (DiaSorin, Saluggia, Italy). Plasma creatinine concentrations were measured by the picric acid colorimetric method, as previously described (Allock et al. 1999). Briefly, saturated picric acid was diluted 10-fold with 1% NaOH. An aliquot of plasma was added to 200 μL of the picric acid/NaOH solution in a microtiter plate for a final volume of 250 μL. Samples were allowed to incubate for 15 min, and the absorbance was read at 490 nm with background correction read at 620 nm. Sodium creatinine was used as a standard. Urine concentration of albumin was determined by ELISA (R&D Systems, Minneapolis, MN) according to manufacturer's specifications. Urine protein concentration was determined by a Bradford -protein assay.

### Renal T lymphocyte immunolocalization

Paraffin-embedded kidneys were sectioned longitudinally into 4-μm-thick sections and mounted on Superfrost slides. Double staining of smooth muscle *α*-actin and T lymphocytes (CD3^+^ cells) was performed using the multiantigen immune staining with Promark system (Biocare Medical, Concord, CA). Following deparaffinization and rehydration, endogenous peroxidase activity was blocked by incubating slides in 3% H_2_O_2_ (Dako, Carpinteria, CA) for 5 min. After rinsing slides with tris-buffered saline (TBS), slides were incubated in antigen retrieval buffer (Dako) in a Black and Decker Steamer (Madison, WI) for 30 min then cooled and rinsed with deionized water then TBS. Rodent Block-R (Biocare, Concord, CA) was applied to slides for 20 min then rinsed with TBS. Slides were incubated in monoclonal smooth muscle *α*-actin (Biocare, 1:100) for 1 h at room temperature then rinsed with TBS. Next, slides were incubated with mouse on rat horseradish peroxidase polymer (Biocare) for 25 min, rinsed with TBS, and stained with diaminobenzidine (Dako) for 6–10 min then rinsed with deionized water. Denaturing solution (Biocare) was applied to slides for 5 min and rinsed with deionized water then TBS. For T lymphocyte staining, anti-CD3 antibody was incubated (Abcam, Cambridge, MA; 1:400) for 60 min then rinsed with TBS. Rabbit on rodent alkaline phosphatase polymer was applied for 25 min then rinsed with TBS. Fast-red substrated chromagen was added for 12–15 min then rinsed with deionized water. Slides were counterstained with hematoxylin (Dako), blued in TBS for 5 min, and rinsed with deionized water. Slides were coverslipped with aqueous mounting media (Shandon; Thermo Scientific, Waltham, MA) and air dried overnight. Numbers of infiltrating CD3^+^ cells in the kidney tissue were determined by counting 10 separate microscopic fields (300 × 300 μm; 40× magnification) in cortex (glomeruli, nonvessel-related and vessel-related areas) and medulla (vessel-related and nonvessel-related areas) in a blinded manner. The final reported numbers are averages of all cell counts per field per area of the kidney.

### Renal histology

Paraffin-embedded kidneys were sectioned longitudinally into 4-μm-thick sections and mounted on Superfrost slides. Structures were visualized with Gomori's Blue Trichrome using bright-field microscopy (Olympus BX40; Olympus America, Melville, NY). Photographs were obtained with a digital camera (Olympus DP12; Olympus America). In a blinded manner, 20 glomeruli per slide each received a glomerulosclerosis score of 0 = 0%, 1 = 25%, 2 = 50%, 3 = 75%, or 4 = 100%. The 20 glomerulosclerosis scores per kidney were then averaged.

The outer perimeter of each protein cast was outlined in digital images of blue trichrome-stained slides, and corresponding areas were calculated using Metamorph software (Molecular Devices, Sunnyvale, CA). Protein cast areas were then normalized to whole outer medullary area.

### Statistical analysis

All data are expressed as mean ± standard error or the mean. Data were graphed and statistically analyzed using a one-way analysis of variance (ANOVA) with GraphPad Prism (La Jolla, CA). Statistical significance was defined as *P* < 0.05.

## Results

### Hemodynamic and activity measurements

**Figure 1:**
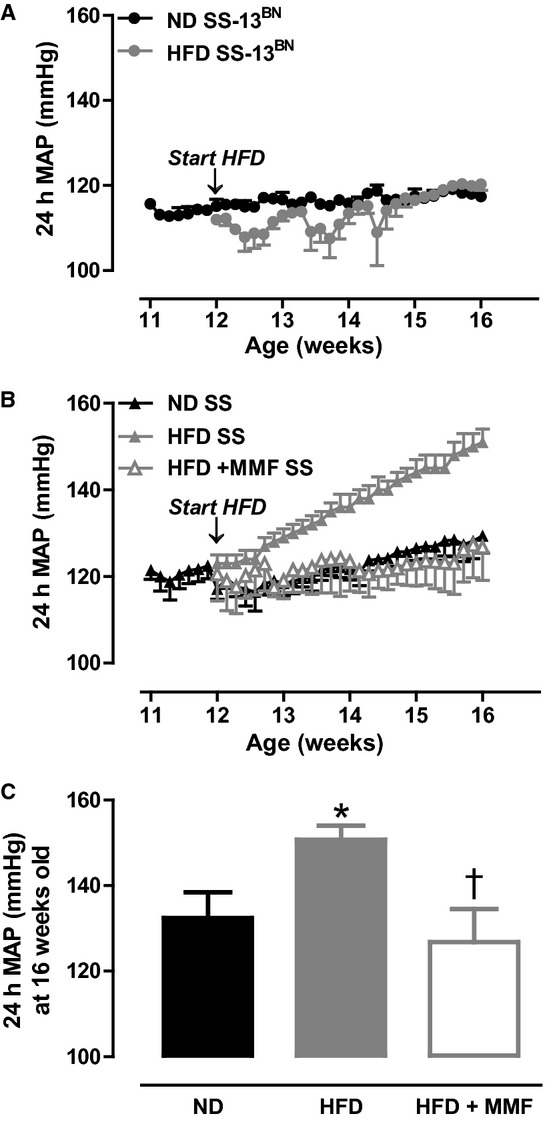
Tracings of 24 h mean arterial blood pressure (MAP) in SS-13^BN^ rats (A, *N* = 4–6) and SS rats (B, *N* = 4–5) on normal diet (ND) or a 4-week high-fat diet (HFD) started at 12 weeks old. A subset of SS rats (*N* = 5) was treated with MMF (30 mg/kg/day, orally in diet) for the duration of HFD. (C) MAP for 24 h in SS rats in the 3 diet groups at 16 weeks old. *P < 0.05 versus ND, ^†^*P* < 0.05 versus HFD.

In the genetic control SS-13^BN^ rats, 4 weeks of HFD did not affect blood pressure (Fig. 1A) or heart rate (beats per minute: ND: 380 ± 4 vs. HFD: 389 ± 13), whereas activity was increased (arbitrary units: ND: 2.3 ± 0.2 vs. HFD: 3.2 ± 0.3, *P* < 0.05). In contrast, in SS rats, 4 weeks of HFD produced hypertension (Fig. 1B and C) and led to greater heart rate values (beats per minute: ND: 295 ± 1 vs. HFD: 380 ± 3, *P* < 0.05) without altering physical activity (arbitrary units: ND: 3.2 ± 0.3 vs. HFD: 2.8 ± 0.3).

When compared to SS rats on HFD alone, chronic treatment with the immunosuppressant MMF during the HFD period prevented HFD-induced hypertension in SS rats (Fig. 1B and C); however, MMF treatment did not affect heart rate (387 ± 6) or activity values (2.8 ± 0.4).

Plasma creatinine was significantly reduced in HFD versus ND SS rats (1.4 ± 0.1 vs. 2.8 ± 0.3 mg/mL, *N* = 6–7, *P* < 0.05), which was restored to ND levels in the HFD + MMF SS rat group (3.1 ± 0.7 mg/mL, *N* = 4).

### Metabolic parameters

Metabolic parameters are detailed in Table 1. Food intake was decreased in the HFD group, whereas Na intake was similar between ND and HFD SS rats as indicated by the comparable Na excretion data between both diet groups (mmol/day: 1.2 ± 0.2 vs. 1.5 ± 0.2, respectively, *N* = 4–5). Body weight along with epididymal and perirenal fat mass were significantly increased in SS rats after 4 weeks of HFD. HFD feeding increased free fatty acids and PRA in SS rats. In contrast, HFD reduced plasma triglycerides, but had little effect on cholesterol and leptin levels.

**Table 1:** Body and fat pad weights and intake parameters at 16 weeks

Parameter	ND	HFD	HFD + MMF
Food intake at 16 weeks old (g/24 h)	24 ± 1 (5)	12 ± 1 (7)*	12 ± 1 (3)*
Body weight (g)	386 ± 10 (10)	454 ± 10 (5)*	415 ± 16 (5)
Epididymal fat (g)	4.5 ± 0.1 (4)	7.7 ± 0.6 (4)*	5.1 ± 0.2 (5)**
Perirenal fat (g)	3.8 ± 0.4 (4)	8.8 ± 0.5 (4)*	4.3 ± 0.5 (3)**
Triglycerides (mg/dL)	118 ± 11 (9)	50 ± 13 (9)*	116 ± 31 (3)
Free fatty acids (μmol/L/L)	25 ± 1 (9)	33 ± 3 (10)*	20 ± 3 (4)**
Total cholesterol (mg/dL)	316 ± 45 (3)	276 ± 27 (3)	275 ± 19 (3)
Very low density lipoprotein (mg/dL)	12 ± 4 (3)	9 ± 3 (3)	9 ± 1 (3)
Low density lipoprotein (mg/dL)	261 ± 33 (3)	227 ± 25 (3)	228 ± 14 (3)
High density lipoprotein (mg/dL)	43 ± 8 (3)	40 ± 0.4 (3)	38 ± 5 (3)
Leptin (ng/mL)	1.5 ± 0.1 (9)	2.2 ± 0.5 (9)	1.8 ± 0.3 (4)
PRA (ng AngI/mL/h)	10 ± 1 (7)	18 ± 2 (7)*	19 ± 2 (5)*
Albuminuria (mg/24 h)	188 ± 26 (4)	109 ± 18 (5)*	91 ± 9 (3)*
Proteinuria (mg/24 h)	190 ± 13 (4)	144 ± 11 (5)*	129 ± 22 (3)*

SS rats were maintained under normal diet (ND) until 16 weeks old or, at 12 weeks, were fed a high-fat diet (HFD) until 16 weeks old. Number of rats is in parentheses. Data analyzed by one-way ANOVA.

* *P* < 0.05 versus ND;

** *P* < 0.05 versus HFD.

MMF treatment did not alter HFD intake or Na intake (1.9 ± 0.5 mmol/day, *N* = 3). In contrast, body weight, epididymal fat mass, and perirenal fat mass were reduced to levels seen in the ND group. Similarly, plasma triglycerides and free fatty acids were restored to ND levels in the HFD + MMF group. Plasma cholesterol levels and leptin did not change in any group. MMF treatment did not alter PRA levels in the HFD groups.

### Renal infiltration of T lymphocytes

**Figure 2:**
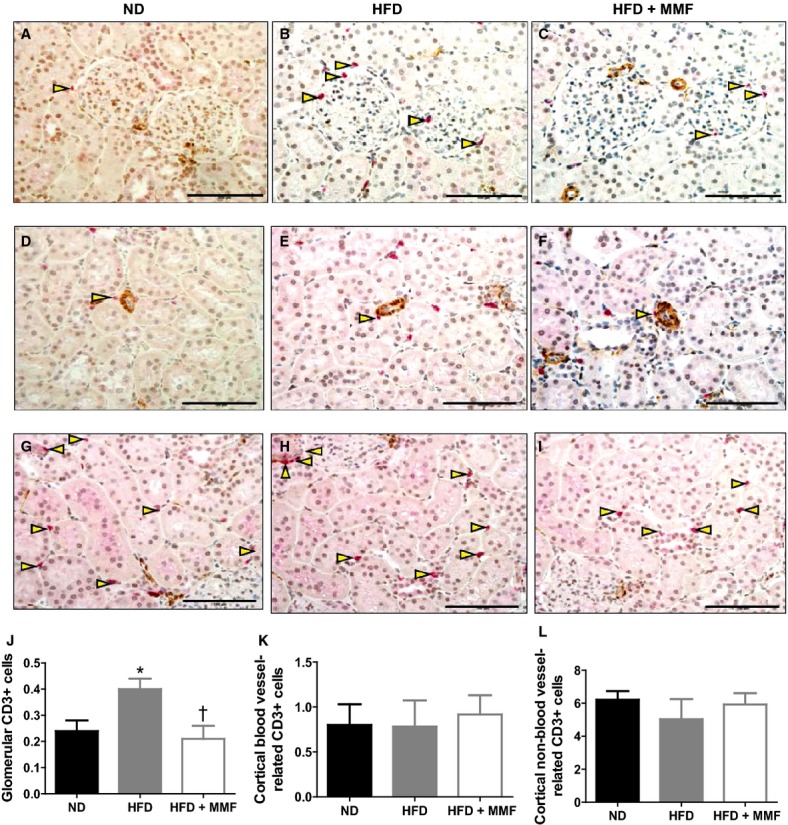
Representative glomerular (A–C, quantified in J) and renal cortical blood vessel-related (D–F, quantified in K) and blood vessel-unrelated (G–I, quantified in L) CD3^+^ cells in SS rats on normal diet (ND) (*N* = 5), high-fat diet (HFD) (*N* = 5), and HFD treated with (+) MMF (*N* = 5). *P < 0.05 versus ND, ^†^*P* < 0.05 versus HFD. Triangles highlight CD3^+^ cells and the bar represents 100 μm.

Renal T-cell infiltration in SS rats on ND, HFD, and HFD supplemented with MMF was examined using immunohistochemistry with double-staining technique to verify localization of T cells within specific renal regions. Representative images of renal glomerular CD3^+^ cells, as a marker of T lymphocytes, are shown in Figure 2A–C. Four weeks of HFD significantly increased glomerular T lymphocyte numbers, and chronic treatment with MMF prevented the HFD-induced increased infiltration (Fig. 2J). We further differentiated between vascular-related and vascular-unrelated CD3^+^ cells in extraglomerular areas of the renal cortex. CD3^+^ cells in the extraglomerular regions of the renal cortex were similar between the three groups of SS rats (Fig. 2D–I and K–L).

**Figure 3:**
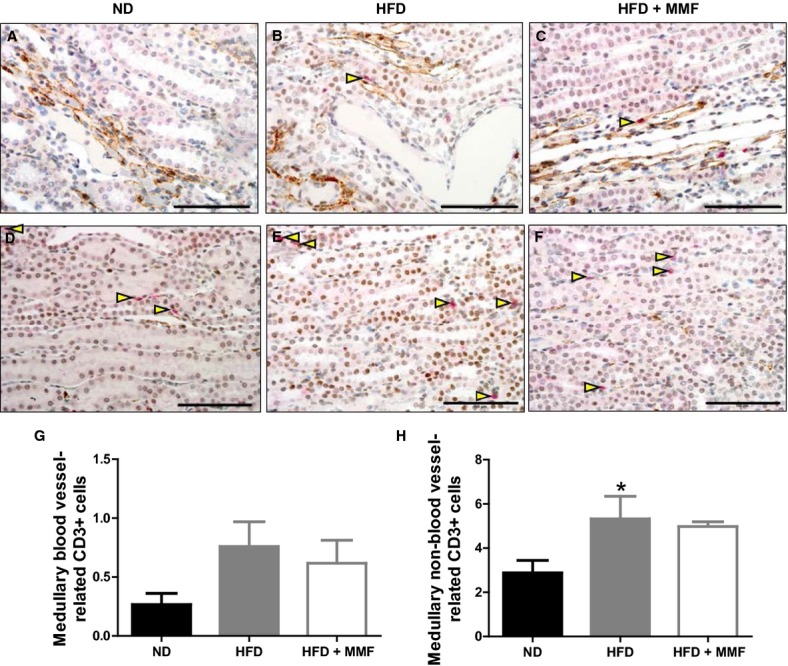
Representative renal medullary blood vessel-related (A–C, quantified in G) and blood vessel-unrelated (D–F, quantified in H) CD3^+^ cells in SS rats normal diet (ND) (*N* = 5), high-fat diet (HFD) (*N* = 5), and HFD treated with (+) MMF (*N* = 5). *P < 0.05 versus ND. Triangles highlight CD3^+^ cells and the bar represents 100 μm.

With regards to HFD effects on the renal medulla, 4 weeks of HFD tended to increase CD3^+^ cells in the vascular-related (Fig. 3G) areas and significantly increased vascular-unrelated (Fig. 3H) CD3^+^ counts compared to ND SS rats. MMF treatment did not alter the numbers of CD3^+^ cells in the renal medulla.

### Renal structural assessment and albuminuria

**Figure 4:**
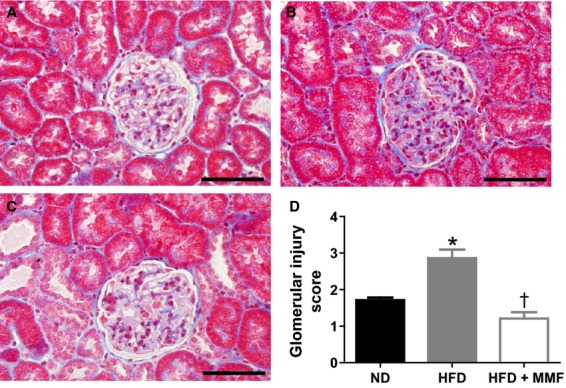
Representative glomeruli from SS rats on (A) normal diet (ND, 43% glomerular injury) (*N* = 5), (B) high-fat diet (HFD, 70% injury) (*N* = 5), and (C) HFD treated with (+) MMF (30% injury) (*N* = 5). *P < 0.05 versus ND, ^†^*P* < 0.05 versus HFD. Bar represents 50 μm.

Representative histological stains of glomeruli from each diet group are represented in Figure 4A–C. Four weeks of HFD was associated with significantly greater glomerular injury in SS rats compared to their ND counterparts (70% vs. 43% glomerular injury score, respectively; Fig. 4D). Chronic treatment with MMF significantly prevented the increase in glomerular injury associated with HFD (30% glomerular injury score; Fig. 4D).

**Figure 5:**
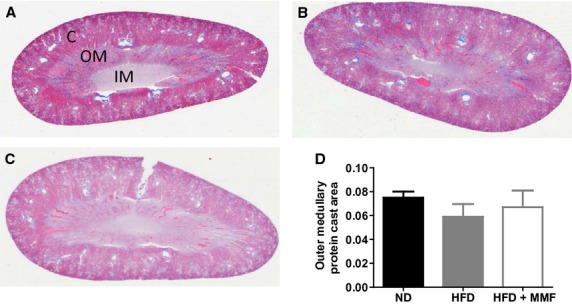
Representative renal medullary protein casts from SS rats on (A) normal diet (ND) (*N* = 5), (B) high-fat diet (HFD) (*N* = 5), and (C) HFD treated with (+) MMF (*N* = 5). Bar represents 100 μm.

Renal protein casts were assessed as a marker of renal tubular injury. The representative histological stains for renal protein casts, which are depicted in Figure 5A–C, illustrate that outer medullary protein cast area was similar in the three diet groups of SS rats (Fig. 5D). No differences in renal vascular injury, tubulointerstitial necrosis, or interstitial fibrosis were detected among the experimental groups.

Interestingly, albuminuria and proteinuria were reduced in SS rats after 4 weeks of HFD compared to ND SS rats (Table 1). MMF treatment did not alter albuminuria or proteinuria (Table 1).

## Discussion

The major findings of this study demonstrate that SS rats are sensitized to HFD-induced hypertension and renal glomerular injury, most likely, mediated through T lymphocytes. A short-term, 4 week, HFD regimen significantly increased mean arterial pressure (MAP), renal glomerular and medullary injury, and renal glomerular and medullary T lymphocyte infiltration. Treatment with the immunosuppressive agent MMF prevented the increase in blood pressure, reduced development of renal glomerular injury coincident with glomerular T lymphocyte infiltration; however, MMF did not alter HFD-induced renal medullary injury or medullary T lymphocyte infiltration.

Nagae et al. (2009) suggested that the SS rat has a susceptibility to develop hypertension with HFD feeding. A subsequent study by Beyer et al. (2012) demonstrated that SS rats have a *bona fide* predisposition to HFD-induced hypertension. In doing so they showed that the genetic control strain for the SS rat, the SS-13^BN^ rat, did not develop the hypertensive response even when fed a HFD 15–20 weeks from weaning. Our results confirm that 4 weeks of HFD induces a blood pressure response in SS rats but not SS-13^BN^ rats. Importantly, although food intake was reduced in the HFD group, salt intake was similar between the groups. These data indicate that the reported changes in MAP were not due to increased consumption of salt in our animals, and confirm a genetic susceptibility to hypertension in the SS rat in response to HFD.

Dahl et al. (1974) showed in transplantation studies that the kidney is critical in the development of hypertension in the SS rat. Extensive evidence implicates renal inflammation in the development of hypertension in the SS rat (Raij et al. 1984, 1985; Ishimitsu et al. 1992, 1994). De Miguel et al. (2011a,b) showed in the SS rat that hypertension, increases in renal T lymphocytes, and renal injury, occurring with either a high-salt diet or a high-protein diet, were prevented by immunosuppressive therapy using tacrolimus or MMF. However, previous studies by our group demonstrated that chronic MMF treatment does not alter blood pressure in a normotensive control rat strain, the Sprague–Dawley rat (Boesen et al. 2010). We speculate that MMF would not alter blood pressure, metabolic profile, or renal CD3^+^ cells infiltration in the kidneys from control SS-13^BN^ rats. Whereas our finding that MMF effectively prevented the hypertensive response to a HFD in SS rats provided impetus to examine specific structures of renal injury and renal localization of T lymphocytes in the Dahl SS strain.

MMF exerts its immunosuppressive actions through inhibition of inosine monophosphate dehydrogenase (IMPDH), the rate-limiting enzyme of the *de novo* synthesis of purines, essential for cell-cycle completion (Allison and Eugui 2000). Due to the lack of an alternative pathway for this synthesis, T and B cells are especially sensitive to MMF. Specifically, it has been shown that MMF is fivefold more potent in inhibiting type II IMPDH, an isoform expressed in activated lymphocytes, than type I IMPDH, present in other cell types (Allison and Eugui 2000). Besides these cytostatic effects of MMF, it has also been shown to induce apoptosis of activated T lymphocytes (Cohn et al. 1999), which could explain the differences that we saw between its actions on glomerular versus medullary inflammation. We speculate that T lymphocytes in the glomerular area are activated and proliferating, and therefore more sensitive to MMF treatment, whereas the ones present in the medulla are not. At this point we are uncertain of what produces this difference in activation status of the T cells in glomerular versus medullary regions of the kidney. An alternative explanation for the different immunosuppressive response to MMF in cortical and medullary areas may be the lack of effect of MMF on decreasing renal injury in the outer medulla, as shown by the lack of change in the medullary protein casts in the three experimental groups.

Along with hypertension and increased numbers of renal glomerular CD3^+^ cells, we found that 4 weeks of HFD induced glomerulosclerosis in SS rats. However, there were no differences in the outer medullary protein cast area or in albuminuria in response to HFD. In support of our findings, Nagae et al. (2009) found that, although blood pressure was elevated following 4 weeks of HFD in SS rats, proteinuria was not elevated. Our findings of increased arterial pressure and reduced albuminuria/proteinuria after 4 weeks of HFD are surprising; however, the most obvious explanation of the decreased protein excretion is the decreased protein consumption in these animals (18% less protein consumed by the HFD groups). This may also explain the lower plasma creatinine found in the HFD group, as dietary protein intake significantly affects serum creatinine concentration (Butani et al. 2002). It is important to note that the animals in the Nagae study were fed a 45% HFD, whereas our animals received a 59% HFD. This difference of ∼15% in the fat content of the diets could be responsible for their lack of glomerular injury even after 8 weeks of HFD and therefore could account for the difference in HFD-induced renal damage between studies.

In our 4-week HFD study, chronic MMF treatment prevented the hypertensive response to HFD and decreased renal T lymphocyte infiltration, a reduction that was only evident in the glomerular area. Intriguingly, albeit in a different organ, it has been shown that HFD induces a shift in the liver T lymphocyte population from antiinflammatory Th2 to proinflammatory Th1 phenotype (Li et al. 2005; Ma et al. 2007). Therefore, although we used a pan-T-lymphocyte marker in this study, we propose that HFD diet induces a proinflammatory Th1 status in kidneys of SS rats.

One of the limitations of the present studies is that we are not able to differentiate if the decrease in glomerular T lymphocytes is due to direct effects on MMF or due to the reduction in arterial pressure that was also observed. Future studies will tease apart the lymphocyte-mediated versus hypertension-mediated renal damage in this model of HFD–induced hypertension. However, we speculate that these effects are dependent on the action of MMF per se, and not secondary to the decrease in blood pressure. In previous studies, De Miguel et al. (2010, 2011a) demonstrated that infiltrating T cells in kidneys of Dahl rats fed a high-salt diet are able to produce angiotensin II and reactive oxygen species (ROS), and, thus, are able to participate in the development and maintenance of hypertension. Similarly, we believe that the direct effects of MMF on glomerular T cells in our studies lead to decreased intrarenal levels of Ang II and ROS, and therefore, decreased arterial pressure. The increased glomerular damage associated with high fat feeding may also be explained by T lymphocyte-derived Ang II and ROS, which have been linked to renal damage (Nishiyama et al. 2004; Lin et al. 2009).

Increased circulating adipose factors like leptin and the renin–angiotensin system, which are classically shown to mediate obesity-induced hypertension in rabbits or classically salt-resistant rats (Dobrian et al. 2001; Iwashita et al. 2002; Lim et al. 2013), do not seem to play a role in our HFD model. Although 4 weeks of HFD elevated PRA in SS rats in our study, MMF prevention of HFD-induced hypertension was observed without a reduction in PRA. Similarly, visceral fat mass and circulating lipids were also increased in response to HFD. Intriguingly, MMF treatment reduced visceral fat pad mass along with circulating free fatty acids. There are pathways that should be examined to explain how adiposity and inflammation mingle to promote HFD-induced hypertension. For instance, it is known that enlarged adipose tissue mass contributes to the elevated circulating free fatty acids in obesity (Bjorntorp et al. 1969). Furthermore, immune cells including T lymphocytes gather in the expanded adipose tissue of obesity (Wu et al. 2007). A study linking T lymphocytes and adiposity demonstrated that mice lacking invariant natural killer T cells have blunted elevations in HFD-induced adipose tissue mass and plasma free fatty acids (Strodthoff et al. 2013). At this point, we are unclear how MMF mediates reductions in the visceral fat pad mass; however, most likely the reduced fat mass also explains the reduced plasma free fatty acids.

The impact of elevated free fatty acids on cardiovascular disease is established. Umpierrez et al. (2009) reported that sustained elevation of free fatty acid levels by intravenous infusion of the fat emulsion Intralipid led to a fast and maintained increased in blood pressure, inflammatory markers, and endothelial dysfunction in a group of diabetic and obese African Americans. Those results obtained in a human population would support the idea that elevated circulating lipid levels are enough per se to develop hypertension and inflammation.

## Perspectives

In conclusion, the present investigation shows that a HFD has deleterious effects on cardiovascular-renal health in SS rats via mechanisms prevented with immunosuppressive therapy. The SS rat is a common model to study genetic susceptibility to cardiovascular-renal disease, similar to the one that certain human populations, such as African Americans, present. Most notably, a link between rat chromosome 13 and SS hypertension has been discovered using this rat strain (Cowley et al. 2001). The finding that SS rats also have an exaggerated blood pressure response to HFD also suggests a genetic mechanism. Reminiscently, genetic studies in spontaneously hypertensive rats implicate chromosome 20 as a link between adiposity, inflammation, and hypertension (Pausova et al. 2003). We speculate that this may predispose SS rats to hypertension and renal disease in response to elevated dietary fat.
